# The liver as an immunological barrier redefined by single‐cell analysis

**DOI:** 10.1111/imm.13193

**Published:** 2020-04-15

**Authors:** Zania Stamataki, Leo Swadling

**Affiliations:** ^1^ Institute of Immunology and Immunotherapy Centre for Liver and Gastrointestinal Research University of Birmingham Birmingham UK; ^2^ NIHR Birmingham Liver Biomedical Research Centre University Hospitals Birmingham NHS Foundation Trust Birmingham UK; ^3^ Division of Infection & Immunity University College London London UK

**Keywords:** immune barrier, liver, liver resident cells, RNA‐seq, single cells, transcriptomics

## Abstract

The liver is a front‐line immune tissue that plays a major role in the detection, capture and clearance of pathogens and foreign antigens entering the bloodstream, especially from the gut. Our largest internal organ maintains this immune barrier in the face of constant exposure to external but harmless antigens through a highly specialized network of liver‐adapted immune cells. Mapping the immune resident compartment in the liver has been challenging because it requires multimodal single‐cell deep phenotyping approaches of often rare cell populations in difficult to access samples. We can now measure the RNA transcripts present in a single cell (scRNA‐seq), which is revolutionizing the way we characterize cell types. scRNA‐seq has been applied to the diverse array of immune cells present in murine and human livers in health and disease. Here, we summarize how emerging single‐cell technologies have advanced or redefined our understanding of the immunological barrier provided by the liver.

## Graphical abstract

The liver is a front‐line immune tissue that plays a major role in the detection, capture and clearance of pathogens and foreign antigens entering the bloodstream through a highly specialized network of liver‐adapted immune cells. Mapping the immune resident compartment in the liver has been challenging because it requires multimodal single‐cell deep phenotyping approaches of often rare cell populations in difficult to access samples. Recently, emerging single‐cell technologies have been applied to the diverse array of immune cells present in murine and human livers, and here we summarize how they have advanced or redefined our understanding of the immunological barrier provided by the liver.

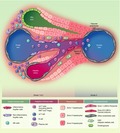

**Abbreviations**
ADME: absorption, distribution, metabolism and excretion
AMNL: amylin liver non‐alcoholic steatohepatitis
BCR: B‐cell receptor
CaHSC: central vein‐associated hepatic stellate cells
cDNA: complementary DNA
CyTOF: cytometry by time‐of‐flight
DEGs: differentially expressed genes
DNA: deoxyribose nucleic acid
ECM: extracellular matrix
FFPE: formalin‐fixed paraffin‐embedded
GFP: green fluorescent protein
HBV: hepatitis‐B virus
HCC: hepatocellular carcinoma
HCV: hepatitis‐C virus
HIV: human immunodeficiency virus
HSCs: hepatic stellate cell
iNKT: invariant natural killer T‐cell
LSEC: liver sinusoidal endothelial cell
MAITs: mucosal‐associated invariant T‐cells
MARCO: macrophage receptor with collagenous structure
MHC: major histocompatibility complex
miR: micro RNA
mRNA: messenger RNA
NK: natural killer
NKT: natural killer T‐cell
RNA: ribonucleic acid
SAMacs: scar‐associated macrophages
scRNA‐seq: single‐cell RNA sequencing
TCR: T‐cell receptor
Treg: regulatory T‐cells
T_RM_: resident memory T‐cells

## Introduction

**Figure 1:**
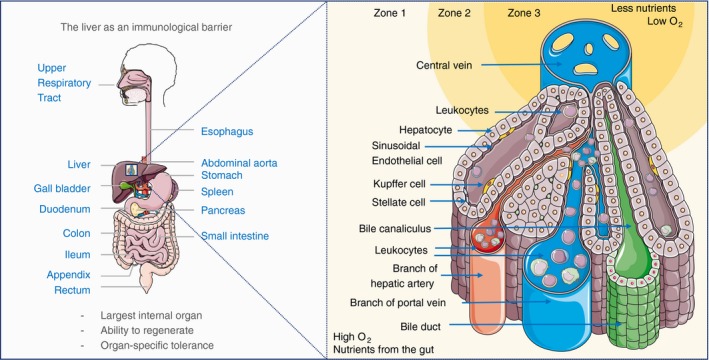
The liver as an immunological barrier. The liver receives a dual blood supply of oxygen‐rich arterial blood from the heart and nutrient/antigen‐rich venous blood, that brings leucocytes that have passed through the gastrointestinal tract, pancreas and spleen. Immune cells from the hepatic artery and portal vein mix in the liver sinusoids and drain into the central vein. The differential access to oxygen and nutrients proximal to the portal triad (hepatic artery, portal vein, bile duct) compared with the oxygen‐ and nutrient‐poor central vein regions affects hepatocyte morphology and forms the basis for defining liver zonation. O_2_, partial pressure of oxygen.

The major portals of entry for pathogens into the body are the skin and mucosal surfaces of the respiratory, urogenital or gastrointestinal tracts. Maintenance of immunological barriers at these sites is an essential component of human health and survival. The liver is a front‐line immune tissue as it receives gut‐draining blood directly from the portal vein, bypassing classic immune sentinel tissues such as the lymph nodes and spleen (Fig. 1).^1^ Thus, a major function of the liver is to detect, capture and clear pathogens and foreign antigens entering the blood via the gut (for review, see Ref. 2). Even when the gut epithelium is damaged, the liver can effectively filter translocating pathogens from the blood, acting as a barrier to systemic infection.^3^ The specialized macrophages of the liver, Kupffer cells, have been recognized for their role in barrier immunity due to their ability to trap and engulf pathogens from the circulation; however, the broader role of liver‐associated immune cells in barrier immunity has not been fully elucidated.

The liver must maintain this immune barrier in the face of constant exposure to foreign but harmless molecules (e.g. food antigens, microbial‐derived products) without eliciting unwanted immune responses. Perhaps for this reason, many immunoregulatory mechanisms are active within the liver at steady‐state and it is considered a largely tolerizing environment, where immune responses are moderated.^4^, ^5^, ^6^ This unique tolerogenic environment is exploited by hepatotropic viruses that develop chronic infections [hepatitis‐B virus (HBV), hepatitis‐C virus (HCV)] and by primary [hepatocellular carcinoma (HCC) and cholangiocarcinoma] and metastatic tumours; however, rapid and robust immune responses can occur under appropriate conditions.^7^, ^8^, ^9^ A fine balance must be struck, as sterile‐liver‐injury results from excessive inflammation in the absence of infection, but chronic infection and cancer result from insufficient immunity.^10^

A highly specialized network of liver‐adapted immune cells is required to integrate local cues, such as metabolites, hormones, cytokines and microbial products to maintain liver homeostasis in health whilst remaining responsive to pathogens. Several recent reviews summarize immunity within the liver, providing detailed information on the immune cell composition, cell−cell interactions, liver residency adaptations, and role of hepatocytes in innate immunity.^2^, ^11^, ^12^, ^13^ Here, we review how emerging single‐cell technologies are strengthening our understanding of the immunological barrier provided by the liver.

## Single‐cell RNA‐seq for mapping heterogeneity of parenchyma and non‐parenchyma in the liver

**Figure 2:**
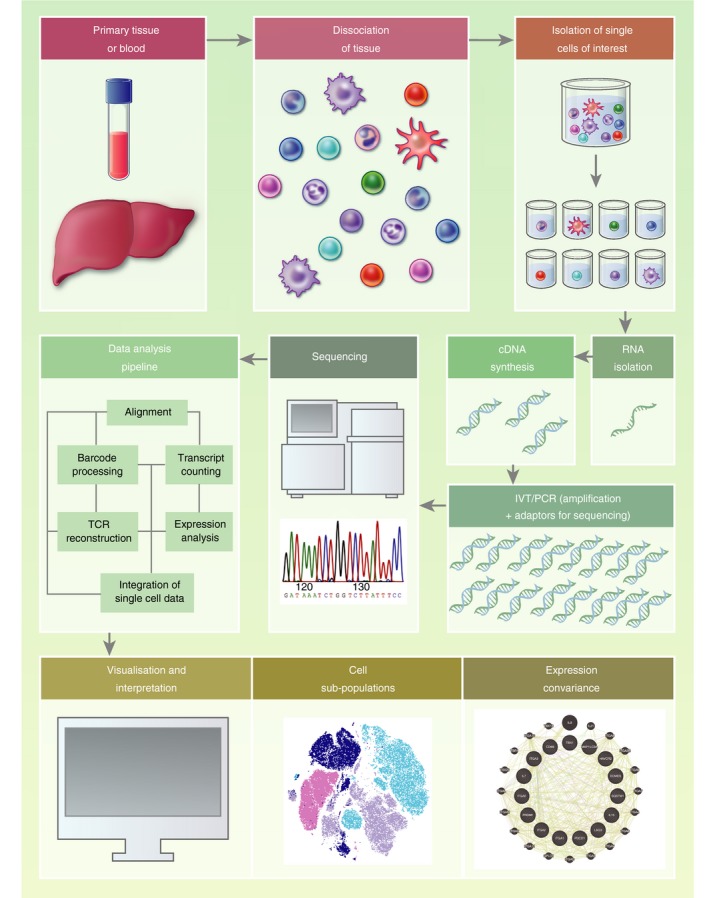
Single‐cell RNA sequencing (scRNA‐seq) experimental workflow. The standard workflow for scRNA‐seq (reviewed in detail in Refs 14, 15) involves: isolation of single cells (commonly by flow cytometric sorting or microfluidics), RNA extraction, enrichment of mRNA [olig‐dT‐enrichment of poly(A) tail mRNA] or depletion of ribosomal RNA (accounting for up to 95% of cellular RNA) before reverse transcription to complementary DNA (cDNA). cDNA is amplified, sequencing adaptors added, before fragmentation to produce a library of short cDNA molecules for sequencing. The resulting sequences are aligned to a reference genome, and the relative quantification of mRNA molecules is calculated for each gene per cell. Statistical modelling is then employed to identify significant differences in the expression level of genes (differential gene expression analysis) between groups of cells and/or samples. cDNA, complementary DNA; PCR, polymerase chain reaction; TCR, T‐cell receptor; IVT, *in vitro* transcription. Example covariance map created using Genemania.^129^

Single cells represent discrete and identifiable entities at which the immune system is fundamentally organized. Much information is lost when we assay the behaviour and functionality of groups of cells as an average, and so assaying individual cells is required to understand their contribution to population‐ and system‐level events. In the absence of single‐cell approaches it is difficult to elucidate the origin of resident and non‐resident immune cell types that mix in the liver microenvironment at the time of sampling. Single‐cell RNA sequencing (scRNA‐seq) allows characterization of cell states, developmental trajectories and key cellular processes using unbiased measurement of the genes expressed by a cell.^14^ The standard workflow for scRNA‐seq is briefly presented in Fig. 2 (for review, see Refs 14, 15). The power of scRNA‐seq is such that work has already commenced on an atlas of the cellular heterogeneity of the human body tissue‐by‐tissue (Human Cell Atlas).^16^ This is now possible due to assays that can simultaneously measure the transcriptome of hundreds of thousands of cells with increasing sensitivity and accuracy.^17^, ^18^

Due to the overall cost of reagents and sequencing, a trade‐off between profiling more cells (breadth) and more transcripts per cell (depth) is considered for each experiment. Selection of the most appropriate scRNA‐seq protocols also depends on the number of samples, cells per sample, sensitivity, and whether full‐length mRNA sequencing or transcript counting is required.^15^ For example, when it is desirable to detect a maximum number of differentially expressed genes (DEGs) on a small number of cells, ‘deep’ single‐cell sequencing is performed (e.g. plate‐based using SMARTseq on 1000–1 000 000 cells often at a depth of 1–6 million reads per cell).^19^, ^20^ Where a larger number of cells need to be assayed, but where identification of lowly expressed genes is not required, microfluidic‐based cell sorting (e.g. 10× genomics platform)^21^ can be used with read depths often limited to 30 000–60 000 reads to restrict sequencing costs. When assaying a large number of cells, costs can be reduced by sequencing along the mRNA transcripts only far enough to ensure accurate identification of the gene it encodes (transcript counting), giving relative mRNA counts, rather than sequencing the full length of each mRNA molecule [which is required for recovering splicing patterns, single nucleotide variants, or immune receptor sequencing of B‐ and T‐cell receptors (BCR and TCR)]. Indeed, many protocols that assay a larger number of cells are incompatible with full‐length mRNA sequencing. With any single‐cell technology, it should be noted that not all mRNA molecules are measured and the data are inherently sparse, but information‐rich, due to the large number of individual data points.

As well as mapping cell composition of individual tissues, scRNA‐seq data are particularly useful for mapping cell differentiation, as subtle coordinated changes in a large number of genes can be used to place each individual cell on a continuum to identify key transitions between cell states.^22^ Fresh human liver tissue access is scarce, and it has proved technically difficult to isolate and obtain single‐cell transcriptomes of fragile liver‐resident cell populations, such as hepatocytes;^23^, ^24^, ^25^, ^26^, ^27^ scRNA‐seq also allows researchers to maximize the unbiased information extracted where cell yields per sample are low, for instance as a result of low viability or rarity of particular cell types, and when the number of samples is limited. Table 1 summarizes the scRNA‐seq studies of human and murine liver discussed below, with references to available datasets and web portals for interrogation of the data provided. Below we review some of the ways scRNA‐seq has advanced our understanding of liver cell types and the immune barrier provided by the liver in health and disease.

**Table 1:** Publicly available scRNA‐seq datasets and web portals from liver samples

First author	Journal	Year	Tissue Samples	Deposited data reference^1^	Web portal
Aizarani^40^	Nature	2019	Human: Whole liver single‐cell suspensions from resected margins	GSE124395	human-liver-cell-atlas.ie-freiburg.mpg.de/
Dobie^55^	Cell Reports	2019	Murine: Mesenchymal cells isolated from *Pdgfrb*‐GFP knockin reporter mouse. Human FFPE liver sections	GSE136103, GSE137720	livermesenchyme.hendersonlab.mvm.ed.ac.uk
Halpern^24^	Nature	2017	Murine (C57BL/6): Isolated hepatocytes	GSE84498	–
Halpern^25^	Nature Biotechnology	2018	Murine (C57BL/6): Sorted single or paired hepatocytes and CD45^+^ or CD32^+^ cells from perfused murine livers	GSE108561	–
Hunter^87^	Journal of Hepatology	2018	Human: Sorted γδTCR^+^ T‐cells (TCR sequence)	SRP113556, SRP096009	–
Krenkel^131^	Cells	2019	Murine (C57BL/6): Sorted non‐parenchymal cells (density gradient) or HSCs from murine livers +/‐ carbon tetrachloride treatment	–	–
Losic^128^	Nature Communications	2020	Human: Multiregional HCC tumour and adjacent non‐tumoral tissue	GSE112271, E‐MTAB‐5905, E‐MTAB‐5899, E‐MTAB‐8127	–
Ma^118^	Cancer Cell	2019	Human: Tumour infiltrating lymphocytes from biopsies of HCC and cholangiocarcinoma	GSE125449	–
Macparland^23^	Nature Communications	2018	Human: Whole liver single‐cell suspensions from potential transplant livers	GSE115469	github.com/BaderLab/HumanLiver
Popescu^110^	Nature	2019	Human: Whole liver single‐cell suspensions sorted into CD45^+/−^ from embryonic and fetal livers	E‐MTAB‐7407	developmentcellatlas.ncl.ac.uk//datasets/hca_liver/
Ramachandran^62^	Nature	2019	Human: Non‐parenchymal (density gradient) liver cells sorted into CD45^+/−^ from liver resections	GSE136103	livercellatlas.mvm.ed.ac.uk
Segal^111^	Nature Communications	2019	Human: Fetal and adult liver, EpCAM/NCAM^+^ from liver resections	GSE130473	–
Tamburini^54^	Frontiers in Immunology	2019	Human: Sorted hepatic lymphatic endothelial cell‐enriched non‐parenchymal cells from liver explants	GSE129933	–
Xiong^88^	Molecular Cell	2019	Murine (C57BL/6): Non‐parenchymal (density gradient) liver cells or bead sorted LSECs, chow or AMLN‐inducing diet	GSE129516, GSE119340, GSE119340,	–
Zhang^121^	Cell	2019	Human: CD45^+^ cells from resected margin, tumour, lymph node, blood and ascites from HCC patients	HRA000069	cancer-pku.cn:3838/HCC/
Zheng^120^	Cell	2017	Human CD3^+^ T‐cells from resected margin, tumour, blood from HCC patients	GSE98638	hcc.cancer-pku.cn

AMNL, amylin liver non‐alcoholic steatohepatitis; FFPE, formalin‐fixed paraffin‐embedded; GFP, green fluorescent protein; HCC, hepatocellular carcinoma; HSCs, hepatic stellate cells; TCR, T‐cell receptor.

^1^ E‐MTAB, access at ebi.ac.uk/arrayexpress; GSE, access at ncbi.nlm.nih.gov/geo; SRP access at ncbi.nlm.nih.gov/sra; HRA accessed at bigd.big.ac.cn/

## Architecture and function of the liver

Single‐cell data need to be considered also in the spatial context of the cells distribution within the liver microenvironment. Figure 1 shows the structural organization of a liver lobule, where immune cells arrive in the portal regions identified by the portal triad. In inflammation, lymphoid aggregates persist in this region and we know little about the kinetics of their egress through the liver. Immune cells from arterial and venous blood coming from the gastrointestinal tract are mixed in a relatively oxygen‐ and nutrient‐rich environment (Zone 1). They then progress through Zone 2 towards the central vein, where hepatocytes are smaller and highly polarized (Zone 3). One can identify the hexagonal structure of a liver lobule by six portal triads in the corners around a central vein. The liver zones are located radially around the central vein, and these can be connected to portal regions by fibrous tissue during prolonged inflammation that leads to bridging fibrosis.

Vascular endothelium restricts immune cells from tissues and, during inflammation, leucocyte rolling, adhesion and extravasation processes are tightly regulated. The liver parenchyma consists primarily of hepatocytes, located behind sinusoidal endothelial layers. Unlike vascular endothelia, which form a barrier to tissues, liver sinusoidal endothelial cells (LSECs) are fenestrated with pores that permit solutes to reach the underlying hepatocytes.^28^ While they permit fluid exchange, LSEC pose a barrier for egressing immune cells and regulate transmigration to the liver parenchyma.

## Mapping hepatocytes

Hepatocytes are epithelial cells that comprise 60%–70% of the liver by mass and 80% by volume, and they play important roles in innate and adaptive immunity.^29^ Early in the acute‐phase response, hepatocytes sense injury signals conveyed by cytokines such as IL‐6, and alter their transcriptional activity to produce acute‐phase proteins such as serum amyloid A, C‐reactive protein, haptoglobin, α1‐antichymotrypsin and fibrinogen. Hepatocytes also secrete IL‐6 and hepatocyte growth factor, which can upregulate the synthesis of albumin, transferrin and fibronectin.

The IL6‐IL6R axis initiated in the acute‐phase response and during hepatocyte injury is an important pathway where hepatocytes can influence liver regeneration, innate and adaptive immunity. As early as 2 hr following hepatocyte trauma, Kupffer cells secrete tumour necrosis factor (TNF)‐α, which triggers hyper‐IL‐6 production, a potent mitogen for hepatocyte proliferation.^30^ IL‐6 long‐term persistence can lead to chronic inflammation and carcinogenesis; however, IL‐6 is also critical to control bacterial, parasitic and viral infection in the liver, such as HBV infection.^31^, ^32^, ^33^ A critical role for IL‐6 was shown in adaptive immunity, where it mediates T follicular helper cell differentiation and germinal centre formation.^34^ Whether hepatocyte‐driven IL‐6 leads to the maturation of liver portal lymphoid aggregates into tertiary lymphoid structures remains to be established.

Rich in innate immune receptors, hepatocytes can sense microbial signals, communicate with innate immune cells and induce myeloid‐derived suppressor cells.^35^ To prevent immune activation to harmless antigens from the gastrointestinal tract, T‐cells that reach hepatocytes through the LSEC pores receive incomplete activation signals and undergo premature apoptosis.^36^ Hepatocytes also scavenge apoptotic cells and cell debris to prevent inflammation, and therefore limit available antigens that could be captured and presented by professional antigen‐presenting cells.^29^, ^37^

Despite their importance in immunity, we know little about hepatocyte functional heterogeneity. Previously we lacked cell surface markers to enable isolation of live zone 1, 2 and 3 hepatocytes in humans (Fig. 1), but it was appreciated that zone 1 hepatocytes perform oxidative functions (fatty acid β‐oxidation, cholesterol synthesis, gluconeogenesis), and zone 3 hepatocytes aid in drug detoxification, glycolysis and lipogenesis.^24^ Recent single‐cell transcriptomic studies have enabled unprecedented characterization of hepatocyte populations (Table 1).

Shalev Itzkovitz’s team have performed the first in‐depth characterization of mRNA expression with spatial resolution of hepatocytes. They identified genes differentially expressed with liver zonation by combining single‐molecule fluorescence *in situ* hybridization with scRNA‐seq.^24^ Up to 50% of genes in mouse hepatocytes were differentially distributed in the liver lobule, an order of magnitude higher than previously estimated.^38^ Identifying cell surface molecules linked to hepatocyte zonation enabled their isolation by FACS for deep phenotyping using CD73, E‐cadherin, size and ploidy measurements, combined with exclusion of CD31^+^ and CD45^+^ endothelial cells and leucocytes, respectively.^39^ In humans, a similar approach can be adopted; however, proteins such as E‐cadherin are less useful to mark periportal hepatocytes, particularly in liver inflammation.^23^, ^40^ By performing scRNA‐seq on single‐cell suspensions of total human liver without enrichment, MacParland *et al.* ^23^ showed that part of this hepatocyte zonation signature was conserved in man in their dataset; however, limited conservation of hepatocytes and endothelial transcriptome‐wide gene zonation was observed by Aizarani *et al.* ^40^

Importantly, the Ben‐Moshe manuscript showed high concordance between protein expression and mRNA measurements, with few notable exceptions (such as HNF4α).^39^ The authors proposed that this may be due to a predominance for spatial regulation of hepatocyte protein levels via transcription or mRNA stability, rather than through translational control or protein stability in the liver. MicroRNA (miR) zonation was also characterized, identifying zonated distribution of important genes such as miR‐122‐5p and miR‐30a‐5p (periportal). Understanding miR distribution is important to decipher hepatocyte contributions to liver development, metabolism, regeneration, liver fibrosis, infection and cancer.^41^, ^42^, ^43^, ^44^, ^45^ Further, spatial identification of metabolic pathways and enzymes using proteomics and *in silico* approaches will inform our understanding of hepatotoxicity, and improve prediction of drug absorption, distribution, metabolism and excretion (ADME).^39^, ^46^, ^47^

Advances in intravital and multiphoton microscopy have revealed intimate associations of lymphocytes with hepatocytes following trans‐endothelial migration, or even through endothelial fenestrations.^48^, ^49^ In these studies investigating T‐cell priming, and others demonstrating unique cell‐in‐cell structures formed when hepatocytes engulfed live T‐cells, the focus has been on periportal hepatocytes.^50^, ^51^ The role of hepatocyte zonation in T‐cell priming and cell‐in‐cell structures remains to be elucidated, and it is an area where spatial single‐cell analyses are highly anticipated.

## Mapping the non‐parenchymal cells of the liver

Several research groups have attempted to broadly map the non‐parenchymal cellular composition of the human liver. As with other cell atlases, a common approach is to apply diverse statistical models to scRNA‐seq data to identify clusters of cells with similar gene expression in an unbiased way, to calculate the gene signature defining those clusters, and then to manually curate the clusters using pre‐existing knowledge of the genes and proteins expressed by known cell types.^14^ For example, Aizarani and colleagues performed scRNA‐seq on single‐cell suspensions of liver resection specimens, and applied their previously described RaceID3 algorithm^52^ to the data to classify cells into 39 discrete subsets.^40^ A key theme identified by this study was the zonation of genes involved in fundamental biological processes across the liver sinusoid in multiple cell types.

**Figure 3:**
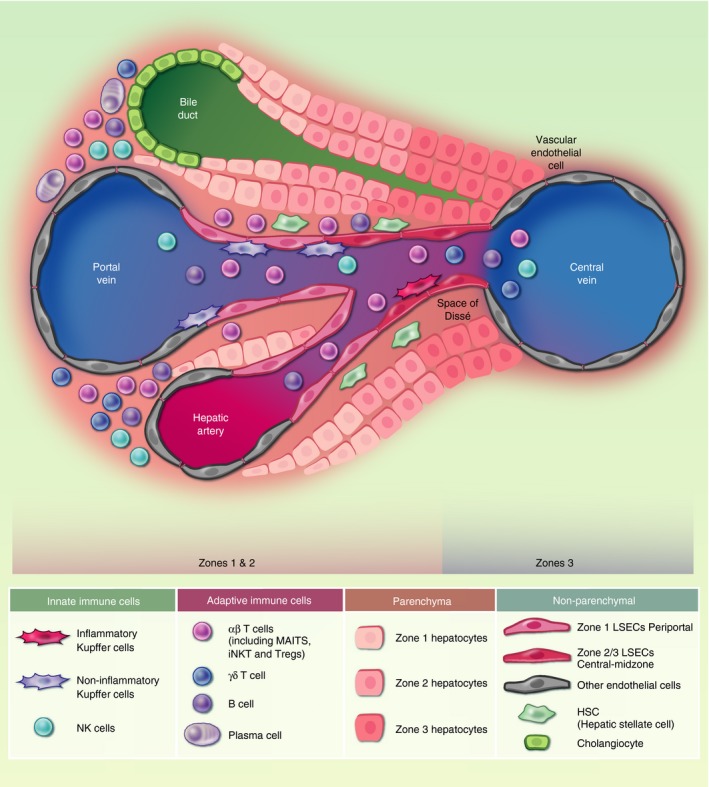
Human liver cell types. Experimental data from single‐cell analyses have provided a broad list of the cell subtypes that are present in the healthy human liver. This schematic shows the location of these parenchymal and non‐parenchymal cell subsets across the portal‐venous sinusoidal axis according to immunohistochemistry and spatial cell sorting where available. Many immune subsets have only been investigated in cell suspension from liver samples without confirmatory spatial staining, as such their locations across this axis are not representative (i.e. αβ‐T‐cells, γδ‐T‐cells, NK cells, B‐cells and plasma cells). Endothelial cells are represented as periportal zone 1 liver sinusoidal endothelial cells (LSECs) and zone 2/3 central‐midzone LSECs,^23^, ^40^, ^53^ and non‐LSEC endothelial cells (representing endothelial cells around the portal vein, central vein, hepatic artery). ‘Inflammatory’ central‐venous Kupffer cells and ‘non‐inflammatory’ periportal Kupffer cells are represented.^23^, ^40^, ^62^ It is important to consider cell−cell interactions between liver‐adapted and liver‐infiltrating cells and hepatocytes. A complex network of cell−cell interactions inform the liver immune barrier, for example LSEC−T‐cell interaction can lead to T‐cell anergy if T‐cells recognize antigen in the absence of co‐stimulation,^28^ and T‐cell−hepatocyte interactions may skew T‐cell responses towards a regulatory phenotype (for review, see Ref. 130). Hepatocytes respond to inflammation by producing immunomodulatory cytokines, such as IL‐6,^30^, ^34^ and can even engulf immune cells, for instance enclysis of CD4^+^ T‐cells,^51^ to influence immune responses in the liver. HSCs, hepatocyte, endothelial cell‐derived signals combine to drive Kupffer cell differentiation;^60^ αβ‐T‐cell, alpha‐beta T‐cell receptor expressing T‐cell; γδ‐T‐cell, gamma‐delta T‐cell receptor expressing T‐cell; LSEC, liver sinusoidal endothelial cells; MAITs, mucosal‐associated invariant T‐cells; NK, natural killer; iNKT, invariant natural killer cell; Treg, regulatory T‐cell.

The highly specialized sinusoidal endothelium makes up a large proportion of the non‐immune parenchymal cells within the liver. LSECs are scavenger endothelia that play an important role in clearance of macromolecules from the blood via clathrin‐mediated endocytosis.^28^ They also act as a barrier to circulating immune cells.^28^ LSECs can activate CD8^+^ T‐cells at high antigen concentrations, leading to the generation of memory cells; conversely, at low antigen concentrations LSECs can mediate T‐cell tolerance and deletion. Immunofluorescence studies had previously defined two types of LSEC by location and phenotype; periportal type I CD36^hi^CD32^−^CD14^−^LYVE‐1^−^ and central‐venous type II CD36^mid‐lo^LYVE1^+^CD32^hi^CD14^+^CD54^+^ (Fig. 3).^53^ scRNA‐seq readily identified two analogous populations of LSECs, as well as a third population of portal non‐LSEC endothelia, and gene signatures for each subset were defined.^23^, ^40^

Using a less harsh cell extraction method, Halpern *et al.* were able to isolate murine hepatocyte‐LSEC doublets for paired‐cell sequencing. By applying the previously described markers of hepatocyte zonation to doublet‐data, they inferred the location of LSECs across the portal‐venous sinusoidal axis and demonstrated zonation in gene expression by liver endothelial cells also.^25^ Using pathway enrichment analysis of the central and midzonal endothelial cells, Aizarani *et al.* ^25^ identified processes that are co‐zonated across the sinusoid, such as common scavenger receptor expression by midzone liver endothelial cells and midzone hepatocytes.

Liver lymphatic vessels also aid the immunological function of organs by draining interstitial fluid, fat, cholesterol and directing immune cells towards the draining lymph nodes. During inflammation of viral and non‐viral origin, lymphatic endothelial cells expand and increase CCL21 expression.^54^ Tamburini and colleagues provided the first single‐cell transcriptomic analysis of liver lymphatic endothelial cells in health and disease. They characterized two lymphatic subsets, including portal endothelial cells (PECs) expressing bone marrow stromal antigen two precursor (BST2), interferon alpha inducible protein 27 (IFI27) and ribonuclease 1 (RNASE1), and fully differentiated lymphatic endothelial cells (LECs) expressing prospero homeobox protein 1 (PROX1), lymphatic vessel endothelial hyaluronan receptor 1 (LYVE‐1), podoplanin (PDPN), vascular endothelial growth factor receptor 3 (FLT4/VEGFR3) and CCL21. They also showed that gene TFF3, which induces expression of VEGF and protects barrier function, was upregulated by the LEC population.

Single‐cell RNA sequencing studies also confirmed and extended the transcriptional signature of hepatic stellate cells (HSCs; also known as perisinusoidal cells or Ito cells), a type of pericyte found in the space of Dissé (between the sinusoids and hepatocytes; Fig. 3). HSCs exist in a quiescent state in healthy liver, and may play a role in antigen presentation, but when activated during liver damage they are the main source of extracellular matrix (ECM)/collagen, therefore contributing to tissue fibrosis (see below).^55^ Transcriptomics of healthy and fibrotic mouse liver also revealed spatial zonation of HSCs across sinusoids.^55^

Single‐cell RNA sequencing experiments have broadly elucidated the heterogeneity within non‐parenchymal cells of the liver, and have highlighted co‐zonation of subsets of hepatocytes, endothelial cells and HSCs across the sinusoid, providing further evidence that diverse cell types show functional co‐operation and/or parallel adaptation to the liver environment (Fig. 3). Further targeted analyses can now be performed to assess the importance of these shared gene signatures in normal liver physiology.

## Innate immune cells at the barrier: Liver resident macrophages

The liver has been described as an organ with ‘predominant innate immunity’ (for review, see Ref. 56) due the large number of innate effector cells present within it. This gland houses the largest collection of phagocytic cells in the body, the majority of which are liver‐resident immobile macrophages, termed Kupffer cells.^57^ Kupffer cells capture pathogens and associated molecules through scavenger and toll‐like receptors, complement and antibody receptors, for example CRIg.^58^ Kupffer cells also act as sentinels, producing chemokines and cytokines to alert other immune cells of infection.^59^ The Guilliams group elegantly demonstrated that stellate cells, hepatocytes and endothelial cells supply the necessary signals to monocytes from the circulation that arrive in the Kupffer cell niche, to differentiate into Kupffer cells in mice.^60^ The same group first used transcriptomics to demonstrate that ZEB2 is a key transcription factor that drives LXRα expression to imprint Kupffer cell identity in mice.^61^ Human Kupffer cells were consistently divided into two major populations by scRNA‐seq: a broadly inflammatory subtype [CD1C^+^ and FCER1A^+^ ^40^; Marco‐(MAcrophage Receptor with COllagenous structure)]^23^ and a non‐inflammatory peri‐portal subtype (Marco^+^ ^23^; TIMD4^+/−^ ^62^; Fig. 3), in accordance with the two subsets described by cytometry in mice.^63^ Integration of human and murine datasets is now required to reach a consensus on Kupffer cell subset definition, and further investigation of their contribution to liver regeneration, fibrosis and liver disease.

## Innate immune cells at the barrier: Natural killer cells and innate‐like T‐cells

Like other barrier sites,^64^, ^65^ the liver is enriched for both natural killer (NK) cells and the innate‐like T‐cells: invariant natural killer cells (iNKT, classic type I NKT), mucosal‐associated invariant T‐cells (MAITs) and γδ‐TCR expressing T‐cells.^2^

Natural killer cells are an innate‐like lymphocyte subset that can regulate both immunity and immunopathology; therefore, it is no surprise that the liver houses the largest population of NK cells in the body.^66^ NK cells screen targets for the absence of self or for signs of infection or tissue injury by integrating signals from a large number of activatory or inhibitory receptors.^66^ The liver contains both recirculating conventional NK cells and an immature‐like liver‐resident population(s) (for review, see Ref. 67). Cytometry by time‐of‐flight (CyTOF) and high‐dimensional flow cytometry have both been applied to single‐cell analyses of liver NK heterogeneity.^68^, ^69^ By interrogating the co‐expression of a panel of proteins that had previously been associated with NK liver‐residency (CD56^bright^ CD16^−^ Eomes^+^ Tbet^+^ CXCR6^+^ CD69^+^ CD49a^+^);^70^, ^71^, ^72^ Filipovic *et al.* ^69^ recently showed that a lack of CD49e expression alone could accurately differentiate liver resident NK from peripheral and recirculating NK.

The two major classes of invariant αβ‐TCR expressing T‐cells, iNKT and MAITs, are both enriched within the liver relative to blood.^73^, ^74^ The invariant αβ‐TCR of iNKT (Vα14‐Jα18 in mice and Vα24‐Jα18 or Vβ11 in humans)^75^ can recognize glycolipid antigens such as α‐GalCer presented on the non‐polymorphic MHC class I‐like molecule CD1d.^76^ Like T‐cells, iNKT can mediate liver injury through cytolysis,^77^ but they have also been implicated in tissue regeneration.^78^ MAITs are the most dominant population of innate‐like T‐cells by magnitude in the human liver,^79^ but they are rare in common laboratory mouse strains.^80^, ^81^ MAITs are responsive to yeast‐ and bacterially derived riboflavin synthesis intermediates presented on major histocompatibility complex class I‐related protein 1 (MR1) through their invariant TCR (Va7.2‐Ja33, with smaller populations of Jα12 or Jα20, preferentially paired with Vβ2 or Vβ13.2 in humans, Vα19‐Jα33 in mice),^79^ and are responsive to innate cytokines such as IL12, IL18 and IFNα, for instance in response to viral infection ^82^. A high frequency of MAITs in the liver may reflect exposure during early life to local riboflavin‐synthesizing commensal bacteria,^83^ but dysbiosis and microbial translocation, for instance resulting from chronic human immunodeficiency virus (HIV) and/or HCV infection, can lead to depletion and dysfunction of MAITs.^84^ The role MAITs play in maintaining the immune barrier of the liver and in liver regeneration are exciting areas of future research.^79^

Also enriched in the liver are γδ‐T‐cells – T‐cells that are selected in the thymus to express a TCR composed of γ and δ chains, which offers a longer more immunoglobulin‐like CDR3 region.^85^ γδ‐T‐cells can recognize protein and non‐protein antigens, in particular phospho‐antigens, directly via their TCR, or through toll‐like receptors and NKG2D.^85^ In mice, the microbial antigens that enter the liver by the portal vein following digestion have been found to sustain γδ‐T‐cells,^86^ which are clonally expanded and display an effector phenotype in the human liver.^87^

When using data on the relative gene expression of all cells within the liver, ‘innate‐like T‐cell’ and ‘NK‐like T‐cell’ populations were identified in all studies;^23^, ^40^, ^62^, ^88^ however, due to their overlapping gene expression there was not the resolution or cell numbers to accurately differentiate subsets within these clusters, despite their known distinct roles in liver immunity (Fig. 3). Targeted enrichment of these cells is required to fully characterize their heterogeneity, and technologies that allow TCR sequencing in parallel to differential gene expression analysis will allow T‐cells with invariant TCRs to be clearly defined (e.g. 10× genomics immune profiling).^21^

## Adaptive immune cells at the barrier: T‐cells and B‐cells

Adaptive immune responses develop days to weeks post‐antigen encounter, but lead to the generation of long‐lived memory cells that mediate rapid recall responses. Over the last decade it has been recognized that an important part of this immunological memory is mediated by highly specialized tissue‐resident populations positioned at barrier sites where pathogen reencounter occurs.^89^, ^90^ Best‐characterized are tissue‐resident memory CD8^+^ T‐cells (T_RM_) that express tissue retention markers, such as CD69, CD103, CXCR3, CXCR6 and CD49a.^91^, ^92^

A detailed characterization of liver‐resident T_RM_ by single‐cell transcriptomic analysis is yet to be performed; however, studies in other tissues provide insight into the role of T_RM_ in tissue immunity_._ Recent work from Donna Farber’s team has used scRNA‐seq to identify three distinct T_RM_ populations in bronchoalveolar lavage of human lung transplant recipients, including donor and recipient cells.^93^ This study demonstrated the survival of donor cells, designated as mature T_RM,_ for over a year in recipients, as well as the ability of recipient T‐cells to infiltrate and re‐seed the tissue following transplantation. As discussed, the liver represents a unique microenvironment for immune cells and, although common phenotypic and transcriptomic adaptations are seen across tissue‐resident T‐cells at different sites,^94^, ^95^ liver‐resident T‐cells also show specific adaptations to this unique microenvironment. We recently reported that liver‐resident CD8^+^ T_RM_ adapt to the liver environment by upregulating their basal rate of autophagy − a cellular pathway that breaks down unwanted cytosolic content, such as damaged mitochondria, and that provides biomolecules from the catabolized content.^96^ Enhanced autophagy could be imprinted on human T‐cells by the prototypical liver cytokine IL‐15 or by IL‐15 producing HSCs. Upregulation of autophagy adapts CD8^+^ T‐cells to combat mitochondrial depolarization (a feature of T‐cells that reside or encounter antigen in the liver),^97^, ^98^ optimize functionality, and acquire tissue residence.^96^ Information on the localization of CD4^+^ and CD8^+^ T_RM_ subsets in the liver, however, is lacking.

Several cell populations are capable of presenting antigen to prime T‐cells in the liver, including liver‐resident dendritic cells, LSECs, Kupffer cells and hepatocytes. The impact of priming by non‐professional antigen‐presenting cells is under intense investigation. An elegant study in mice showed that T‐cell priming by Kupffer cells leads to seeding of the liver with immotile functional effector T‐cells;^49^ in contrast, when antigen was presented by hepatocytes, the natural target of the hepatotropic viruses such as HBV and HCV, T‐cell responses with poor effector function were generated. Several murine studies have investigated the impact of the level and persistence of antigen and the type of presenting cell on the induced T‐cell response, showing that dysfunctional or tolerized T‐cells are common outcomes of liver‐priming.^99^, ^100^, ^101^, ^102^

Single‐cell transcriptomic analyses are also lacking for liver B‐cell populations, despite the mounting evidence for the role of B‐cells in immunity to HBV and HCV infection, and an emerging role in cancers.^103^, ^104^, ^105^ We recently characterized intrahepatic HBV‐specific B‐cells in patients chronically infected with HBV, showing that they were predominantly dysfunctional T‐bet^+^ atypical memory B‐cells that expressed the inhibitory receptor PD‐1.^106^, ^107^ In our estimation, B‐cell subset localization in combination with deep phenotyping, for example by mass cytometry as in Ref. 108 is needed to address the contribution of B‐cell populations to the immune barrier provided by the liver.

## What we can learn from the fetal liver

Cellular constituents of the blood and immune systems develop during early embryogenesis, and the fetal liver functions as the major organ of haematopoiesis prior to fetal bone marrow development.^109^ Popescu *et al.* ^110^ used scRNA‐seq to comprehensively map the developing human fetal immune network during early embryogenesis and, importantly, validated the full spectrum of cell states identified using a manually curated cytometry panel of just 48 surface markers. Inferred trajectories of haematopoietic development suggested the central haematopoietic stem cell node gave rise to three lineages: B‐cells and innate or T‐lymphoid cells, myeloid cells and megakaryocyte–erythroid–mast cells, with γδ‐T‐cells and αβ‐T‐cells sequentially seeding the fetal liver following their exit from thymus. Myeloid progenitors, monocytes, macrophages and dendritic cell clusters were all present in the fetal liver.^110^, ^111^ Overall, this work highlights the important role the fetal liver plays in haematopoiesis and the development of the immune system.

## Breakdown in barrier and change in intrahepatic lymphocytes in disease: Inflammation and fibrosis

Adult organ function is normally retained through tissue repair rather than regeneration; however, the liver is an exception to this as it is endowed with impressive regenerative capacity, with a third of murine hepatocyte mass regenerating in ~10 days after two‐thirds partial hepatectomy.^10^ scRNA‐seq is being applied to identify cells of a progenitor nature that can potentially imbue the liver with regenerative capacity, for instance, the existence of a bipotent liver progenitor cell arising from bile duct cells has gained weight from trajectory analyses and liver organoid development.^40^, ^111^

Regeneration of the liver involves hepatocyte and endothelial cell proliferation and remodelling of the ECM by stromal cells. Repetitive injury and persistent inflammation prolongs this process, leading to an abnormal state known as fibrosis, in which excessive ECM accumulates.^112^ As the number of functional hepatocytes and overall liver function is reduced, and the liver architecture and blood supply are disturbed, fibrosis can develop into cirrhosis and end‐stage liver disease, culminating in liver failure. Different diseases manifest from progressive liver damage, and the importance of the liver as an immunological barrier becomes clear as infection is a major cause of death for patients with end‐stage liver diseases.^113^ Patients with cirrhosis are at increased risk of infections owing to impaired immunity and a propensity for bacterial translocation from the gut, especially in the setting of portal hypertension.^114^

The major cellular players and pathways involved in liver fibrosis have also been investigated recently by single‐cell approaches. Using a combination of trajectory analysis and functional assays, a complex interplay between multiple cells of the lymphocyte, endothelial and mesenchymal lineages was uncovered during development of liver fibrosis. By assaying gene expression across mesenchymal cells in healthy and fibrotic murine livers, Dobie *et al.* ^55^ identified central vein‐associated HSCs (CaHSCs) as the dominant pathogenic collagen‐producing cells during centrilobular injury‐induced fibrosis in mice. Within the gene signature of CaHSCs, lysophosphatidic acid receptor 1 (*LPAR1*) was highlighted as a potential therapeutic target.

Single‐cell analysis has also helped to identify a circulating population of scar‐associated macrophages (SAMacs) in both a mouse model of NASH^88^ and in cirrhotic human livers.^62^ SAMacs are pro‐fibrogenic macrophages able to induce fibrillar collagen expression by HSCs, and their frequency correlates with fibrosis scores in patients with NASH.^62^ ACKR1^+^ and PLVAP^+^ endothelial cells and a subset of PDGFRα^+^ collagen‐producing mesenchymal cells were also indicated as a major expanded population in cirrhotic livers.^62^, ^115^ Importantly, through comprehensive modelling of the potential ligand‐receptor interactions (CellPhoneDB;^116^ cellphonedb.org) between cells in the liver fibrotic niche, several targetable pro‐fibrogenic pathways have been highlighted (TNFRSF12A, PDGFR and NOTCH signalling).^62^ Finally, although the involvement of lymphocytes in fibrosis was not extensively investigated in Ramachandran *et al.*,^62^ it is clear that subsets of CD4^+^ T‐cell, CD8^+^ T‐cells and NK cells are reduced in cirrhosis. As with other scRNA‐seq datasets, there is a wealth of data emerging that can be re‐purposed to focus in on different immunological questions, such as the nature of these lymphocyte subsets, and how they shape or are shaped by ongoing fibrosis. Several key cell populations and pathways have been identified by single‐cell analyses that may allow pathology‐based patient stratification and that can be investigated as therapeutic targets for a range of fibrotic diseases.

## Breakdown in barrier and change in intrahepatic lymphocytes in disease: Cancer

The liver is a common site for both primary and metastatic tumours and, with HCC‐related mortality predicted to rise by 38% by 2035 in the UK, HCC is a tumour of unmet clinical need.^117^ scRNA‐seq has been applied to cancer samples from the liver to study tumour‐infiltrating immune cells and tumour transcriptomic diversity. Primary liver cancer can arise spontaneously, but predominantly results from chronic liver inflammation of various origins, including: viral infection, autoimmunity and metabolic injury, such as fatty diet or alcohol abuse. It comprises HCC and cholangiocarcinoma, which arise from malignant transformation in hepatocytes or cells of the bile duct, respectively. Ma *et al.* ^118^ compared these cancers by droplet‐based scRNA‐seq and showed that more diverse tumours had the poorest prognosis, which was also supported by Zhang *et al.* ^119^ for HCC. It is somewhat surprising that cancer can flourish in a barrier site with ample immune defences; however, as previously mentioned, the liver moderates immune responses and contains high frequencies of regulatory immune cells compared with blood. Single‐cell transcriptomic data revealed that the proportion of regulatory T‐cells in particular was linked to poor prognosis in HCC.^119^, ^120^, ^121^

A seminal study by Zheng *et al.* ^120^ analysed 5063 single T‐cells isolated from peripheral blood, tumour and adjacent normal tissues from six HCC patients. This work demonstrated in unprecedented depth the phenotype of T‐cells in HCC, identifying discrete populations of activated LAYN^+^ regulatory T‐cells and exhausted LAYN^+^ CD8^+^ T‐cells in these patients. Due to the random nature of TCR generation, through recombination of *variable*, *diversity* and *joining* genes, TCR sequences can be used as unique identifiers of T‐cell clonality.^122^ Zheng *et al.* ^122^ mined their scRNA‐seq data to bioinformatically reconstruct full‐length TCR α and β chain sequences for T‐cells from five HBV‐positive HCC patients. They demonstrated increased numbers of clonally expanded TCRs in tumour‐associated T‐cells compared with blood, suggesting local proliferation.^120^ Expanded clonotypes were more likely to have an exhausted gene signature, and some clonotypes were present within both the exhausted T‐cell cluster and the CD8^+^FOXP3^+^ regulatory T‐cell (Treg) cluster, showing divergent differentiation.^120^ These data, combined with that from a growing number of other solid tumours,^123^, ^124^ have contributed to the identification of a common gene expression signature of tumour‐associated regulatory T‐cells;^125^ this hints at global features of Treg modification in tumours that could be targeted therapeutically.

Immunohistochemistry and CyTOF experiments revealed distinct tumour‐infiltrating immune cell profiles in HCC tumours of HBV or non‐viral origin.^126^ Regulatory T‐cells and T_RM_ were enriched in HBV‐related HCC, whereas Tim‐3^+^ CD8^+^ T‐cells and CD244^+^ NK cells were enriched in non‐viral tumours. Further, transcriptomics and *in vitro* T‐cell proliferation assays showed functional differences between the T‐cell populations, with HBV‐associated T‐cells being functionally exhausted when compared with T‐cells infiltrating non‐viral HCC.^126^

Single‐cell approaches characterizing the tumour microenvironment in liver cancer have the potential to inform the design of immunotherapy approaches and may provide a measure of their efficacy. In the context of checkpoint inhibitor treatments for various solid tumours, including liver cancer, a proportion of patients reported benefit,^117^ but some also develop autoimmune‐like pathology.^127^ Checkpoint‐inhibitor induced hepatitis is a serious complication that can lead to acute liver failure, characterized by high CD8^+^ T‐cell infiltrate. High‐dimensional characterization of the liver immune compartment will help us to understand liver toxicity in these patients; to this end, a recent study by Losic *et al.* ^128^ combined RNA, DNA and TCR sequencing and single‐nucleotide polymorphism array data across multiple regions of liver tumours to map spatio‐temporal interactions between cancer and tumour‐infiltrating lymphocytes. The authors used a powerful combination of metrics for tumour‐infiltrating lymphocyte burden and clonality, regional neoepitope variance and potential viral cofactor signals by RNA‐seq to suggest that tumour neoantigen rather than viral antigen may drive immune cell recruitment. This combination of single‐cell approaches provided the most in‐depth set of experiments towards understanding tumour heterogeneity and its relationship with infiltrating immune cell recruitment and persistence in tertiary lymphoid structures.

## Summary of the liver as an immunological barrier redefined by single‐cell analyses

The existence of highly specialized immune cell subsets that are adapted to reside long term in tissues is now well supported by experimental data. To understand their contribution to immunity and pathology during steady‐state and disease, immune cells must be studied in tissues and at the site of disease. Single‐cell transcriptomic studies have markedly improved our understanding of the complexity of the parenchymal and non‐parenchymal compartments within the liver, and has laid the foundations for an atlas of the liver to be constructed. Figure 3 represents a framework of our current understanding of the cellular composition of the liver, informed by recent scRNA‐seq studies listed in Table 1. We can now employ targeted experiments to add layers of detail to this atlas, allowing perturbations in disease to be more readily identified and targeted.

Single‐cell RNA sequencing offers a static, descriptive snapshot of the transcriptome; however, advances in wet lab assays and analytical pipelines will allow additional layers of information to be superimposed on gene expression data (spatial organization, proteome, metabolome, genome, etc.), and the ontogeny and lineage relationships of cells to be investigated. In addition to limitations in depth and sensitivity driven by transcript expression levels, the plasticity of immune cells makes the definition of immune subsets complex. With the unprecedented resolution of single‐cell analyses, it is clear that the problem we now face is in identifying biologically meaningful differences and in agreeing on consensus definitions of cell subsets so that data can be compared across studies.

A wealth of publicly available data is becoming available, which can be reanalysed and reinterpreted, adding extra value to these experiments (Table 1). Further functional validation and targeted approaches are needed, as is exemplified by the inability of ‘NK‐like subsets’ to be differentiated when small numbers of cells are analysed in single‐cell suspensions of total liver.^23^, ^40^, ^62^, ^88^ As represented in Fig. 3, spatial single‐cell analyses are highly anticipated for all immune subsets in the liver. We now have the tools to examine the immune system in unprecedented detail and, as these approaches become more sensitive and cost‐effective, they will reveal new ways to understand and treat liver diseases and liver cancer.

## Disclosures

The authors declare no conflict of interest.
